# Neutrophil-to-prognostic nutritional index ratio and short-term mortality in heart failure: an exploratory association without demonstrated superiority over PNI, NLR, or SII

**DOI:** 10.3389/fnut.2026.1995940

**Published:** 2026-10-23

**Authors:** Zhenyu Yuan, Mohan Li, Lei Zhao, Hong Geng, Qiang Ji, Kun Zuo, Yuanzhi Luo, Guanli Liang, Gengxu He

**Affiliations:** 1 Department of Thoracic and Cardiovascular Surgery, The First Affiliated Hospital of Hebei North University, Zhangjiakou, China; 2 Department of Pediatrics/Neonatology, Hebei General Hospital, Shijiazhuang, China

**Keywords:** all-cause mortality, heart failure, illness severity, MIMIC-IV, NPNR, systemic inflammation

## Abstract

**Background:**

Systemic inflammation and greater illness severity are associated with adverse short-term outcomes in heart failure (HF). The neutrophil-to-prognostic nutritional index ratio (NPNR) combines neutrophil count with the prognostic nutritional index (PNI), a composite derived from serum albumin and lymphocyte count that is influenced by inflammation and acute illness. This study examined the association of NPNR with 28-day and 90-day all-cause mortality in hospitalized patients with HF and assessed its replication in MIMIC-IV.

**Methods:**

The analysis included 1,885 patients with calculable NPNR from the Zigong Heart Failure Database and 1,150 adult ICU patients with HF from MIMIC-IV. Log2-transformed NPNR was examined using Cox regression, Kaplan–Meier analysis, restricted cubic splines (RCS), and sensitivity analyses, including parsimonious and Firth-penalized Cox models. MIMIC-IV provided an independent clinical setting for assessing the direction of the association.

**Results:**

In Zigong, each doubling of NPNR was associated with higher 28-day mortality (HR 1.728, 95% CI 1.130–2.642, *p* = 0.012) and 90-day mortality (HR 1.684, 95% CI 1.124–2.524, *p* = 0.012). However, the expanded models included only 32 and 36 deaths, respectively, and the tertile-based estimates had wide confidence intervals. RCS analyses suggested nonlinearity at both time horizons. NPNR AUCs were 0.727 and 0.717, with no significant superiority over PNI, NLR, or SII in DeLong comparisons. In MIMIC-IV, the continuous association was borderline significant at 28 days (HR 1.112, *p* = 0.049) and not statistically significant at 90 days (HR 1.081, *p* = 0.096). Higher NPNR tertiles were associated with greater mortality, while RCS analyses showed no clear nonlinearity.

**Conclusion:**

Higher NPNR was associated with short-term all-cause mortality in the Zigong HF cohort, with weaker evidence in the MIMIC-IV ICU cohort. NPNR may be considered an exploratory laboratory-derived marker associated with systemic inflammation and illness severity. It did not demonstrate superior discrimination over PNI, NLR, or SII, and its incremental prognostic value remains uncertain. These findings do not support its routine use as a clinical decision tool.

## Introduction

Heart failure (HF) is a complex clinical syndrome resulting from structural or functional cardiac abnormalities and remains associated with a substantial disease burden and mortality risk, particularly in the context of global population aging (1–4). For hospitalized patients with HF, the period during hospitalization and shortly after discharge represents a high-risk “vulnerable phase,” during which short-term outcomes are influenced not only by cardiac pump function and hemodynamic status but also by renal function, comorbidities, systemic inflammation, congestion, and illness severity (5). Changes in serum albumin and circulating leukocyte counts may accompany inflammation, congestion, and acute clinical deterioration without necessarily indicating nutritional status. Routine laboratory indices incorporating these measurements may therefore be associated with short-term outcomes, even when their components are not disease-specific.

Systemic inflammation is an important component of HF pathophysiology. Innate immune activation, cytokine signaling, immune-cell trafficking, and interactions between the heart and peripheral tissues contribute to disease progression (6–11). Neutrophils are key effector cells of innate inflammation and may participate in endothelial dysfunction, microcirculatory impairment, and tissue injury through protease release, oxidative stress, cytokine production, and neutrophil extracellular trap formation (12). Clinically, a higher neutrophil-to-lymphocyte ratio (NLR) has been associated with adverse outcomes in HF (13), and composite indices such as the systemic immune-inflammation index (SII) have also been investigated for prognostic assessment (14). These findings support the relevance of inflammatory burden in HF, while circulating leukocyte and albumin profiles may also vary with comorbid conditions and the severity of acute illness.

The prognostic nutritional index (PNI), derived from serum albumin and peripheral lymphocyte count, is a laboratory-derived composite associated with inflammatory and systemic illness burden. Lower PNI has been associated with increased mortality in acute HF (15), and subsequent meta-analyses have supported its prognostic relevance across HF populations (16). Serum albumin, a major component of PNI, is a negative acute-phase reactant with a relatively long half-life. Its concentration may be affected by inflammation, hepatic synthesis, renal dysfunction, congestion, and fluid balance, and low albumin has been associated with adverse cardiovascular and HF outcomes (17, 18). Although its conventional name refers to nutrition, PNI cannot establish nutritional status in acutely ill patients; its prognostic association may partly reflect inflammation and overall illness severity. Recent HFpEF research has also examined the neutrophil percentage-to-albumin ratio as an inflammatory index associated with diuretic resistance and mortality (19). These observations support studying combined neutrophil–albumin markers, without establishing biological independence or superiority over their components.

The neutrophil-to-prognostic nutritional index ratio (NPNR) is calculated as neutrophil count divided by PNI and combines laboratory components that may be influenced by inflammatory and illness-related processes. Li et al. reported an association between NPNR and long-term mortality after acute myocardial infarction in Chinese and US cohorts (20), suggesting possible prognostic relevance across cardiovascular settings. However, its association with short-term outcomes in HF, the shape of the exposure-response relationship, and its reproducibility across clinical environments remain unclear. Accordingly, we used the Zigong Heart Failure Database to evaluate NPNR in relation to 28-day and 90-day all-cause mortality and examined replication in an ICU HF cohort from MIMIC-IV. Analyses focused on the continuous association, dose–response gradient, and exposure-risk pattern, together with comparisons against commonly used inflammatory and albumin-based indices.

## Materials and methods

### Study design and cohorts

This retrospective observational study included a Chinese hospitalized HF discovery cohort and a US ICU HF external replication cohort. The discovery cohort was derived from the Zigong Heart Failure Database, established using electronic health records at the Fourth People’s Hospital of Zigong, Sichuan Province, China. It comprised consecutive patients hospitalized with HF between December 2016 and June 2019, with externally collected outcomes at 28 days, 3 months, and 6 months (21, 22). The original database contained 2,008 patients. The replication cohort was drawn from MIMIC-IV version 3.0, which contains de-identified hospital and ICU electronic health records from Beth Israel Deaconess Medical Center covering 2008–2022 (23, 24). Reporting followed the STROBE recommendations for observational studies (25).

The Zigong database contained 2,008 hospitalized patients with HF. Calculation of NPNR required neutrophil count, lymphocyte count, and serum albumin; these components were missing in 27, 27, and 102 patients, respectively, with overlapping missingness. Exclusion of 123 patients lacking at least one component left 1,885 patients with valid NPNR values. Cohort-specific tertiles comprised T1 (*n* = 629), T2 (*n* = 628), and T3 (*n* = 628) (Figure 1). The 28-day time-to-event analyses included 1,885 patients and 32 deaths. At 90 days, vital status was available for all 1,885 patients, including 37 deaths; one death lacked an exact event time, leaving 1,884 patients and 36 deaths for Cox, Kaplan–Meier, and RCS analyses. A fixed complete-case sample was used across adjusted Cox models at each time horizon.

**Figure 1:**
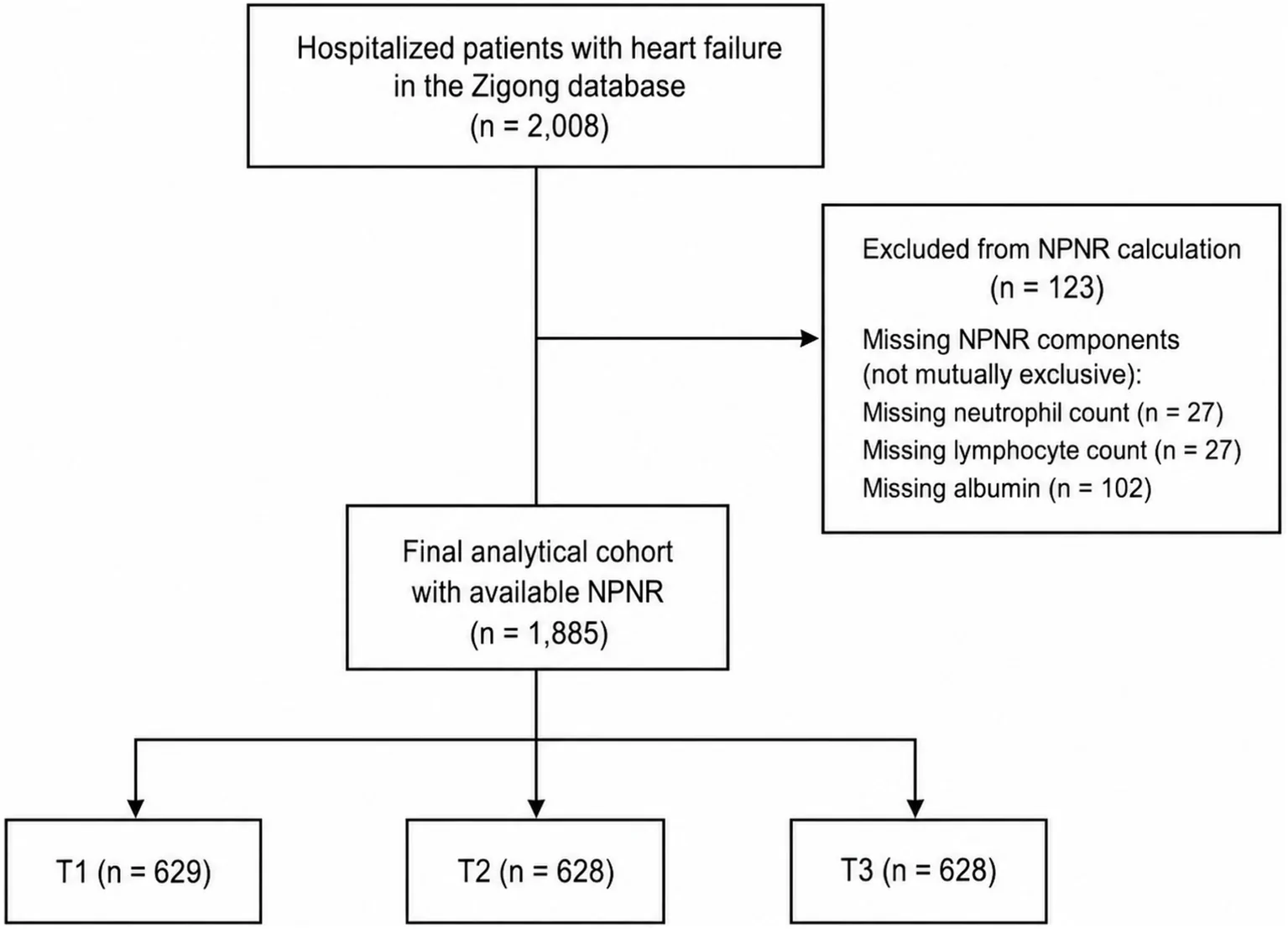
Flowchart of participant selection in the Zigong discovery cohort. Patients with at least one missing NPNR component were excluded; component-specific missingness was not mutually exclusive. The final cohort was divided into cohort-specific NPNR tertiles.

The MIMIC-IV cohort was based on a previously constructed dataset of adult ICU patients with HF. The initial file contained 1,182 records. Retaining the earliest eligible ICU stay per patient yielded 1,150 unique patients with available NPNR components and main model covariates. This cohort was used to examine whether the direction and gradient of the NPNR association were reproducible across clinically distinct populations, rather than to formally validate the performance of a prediction model. Replication analyses included baseline characteristics, Cox regression, Kaplan–Meier curves, and RCS.

### Exposure, outcomes, and covariates

PNI was calculated as serum albumin (g/L) + 5 × lymphocyte count (10^9^/L), and NPNR as neutrophil count (10^9^/L)/PNI. For MIMIC-IV, albumin was converted from g/dL to g/L before index calculation. Laboratory measurements were obtained during the first day of hospitalization in Zigong and the first day of ICU admission in MIMIC-IV. Given its right-skewed distribution, NPNR was primarily analyzed on a log2 scale, so the Cox HR represents the association per doubling of NPNR. Tertiles were formed separately within each cohort (approximate cut points 0.095 and 0.143 in Zigong; 0.206 and 0.371 in MIMIC-IV) to describe within-cohort risk gradients rather than transferable clinical thresholds. PNI, the neutrophil-to-lymphocyte ratio (NLR), and the systemic immune-inflammation index (SII) were also calculated in Zigong for fixed-horizon discrimination comparisons. PNI and NPNR were treated as laboratory-derived indices and were not used to determine nutritional status.

The primary outcome was 28-day all-cause mortality, and the secondary outcome was 90-day all-cause mortality in both cohorts. Fixed-horizon 180-day mortality was additionally evaluated as a Zigong sensitivity outcome. Continuous log2(NPNR) was the primary exposure in progressively adjusted Cox models. The crude model included NPNR alone; Model 1 adjusted for age (>79 versus ≤79 years) and sex; Model 2 added the Charlson Comorbidity Index (CCI) and stratified the baseline hazard by NYHA functional class; and Model 3 additionally included standardized ln(BNP), standardized estimated glomerular filtration rate (eGFR), and standardized serum sodium. Age is recorded in eight intervals rather than exact years in the public Zigong dataset. The >79-year grouping limited the number of model parameters, while the original eight ordered age intervals were retained in a sensitivity analysis. A four-parameter core model containing NPNR, age group, sex, and NYHA class IV versus II/III, together with corresponding Firth-penalized models, provided further assessments of robustness. Tertiles were used primarily to describe the risk gradient, with emphasis placed on continuous NPNR estimates.

In MIMIC-IV, Model 1 adjusted for age (>79 versus ≤79 years) and sex; Model 2 additionally adjusted for CCI and standardized systolic blood pressure; and Model 3 additionally adjusted for standardized eGFR and serum sodium. A sensitivity analysis used continuous standardized age in place of binary age, as numeric age was available in this cohort. Adjustment sets differed between cohorts because of their clinical settings and available variables. Cross-cohort comparisons therefore focused on the direction and pattern of associations rather than treating differences in HR magnitude as proof of distinct biological effects.

### Statistical analysis

Continuous variables were summarized as mean ± standard deviation or median (interquartile range), as appropriate, and categorical variables as number (percentage). Baseline characteristics were described by NPNR tertile in both cohorts. Between-group *p*-values were interpreted descriptively rather than used for covariate selection; standardized mean differences (SMDs) were also reported in Zigong, together with the missingness of selected variables.

Time-to-event associations were estimated using Cox proportional hazards regression, with hazard ratios (HRs), 95% confidence intervals (CIs), and two-sided *p*-values. T1 served as the reference in tertile analyses, and trends were tested using the median NPNR value of each tertile as a continuous score. The proportional hazards assumption in Zigong was assessed using scaled Schoenfeld residuals and global tests; tertile-specific survival was compared using Kaplan–Meier curves and log-rank tests. In Zigong, events per parameter were calculated from the number of estimated regression coefficients; NYHA stratification permitted separate baseline hazards without adding NYHA regression coefficients. Firth-penalized Cox models were used to examine small-event sensitivity (26).

Restricted cubic splines (RCS) used knots at the 10th, 50th, and 90th percentiles of NPNR, with the median as the reference. Zigong RCS models included Model 3 covariates and NYHA stratification, whereas MIMIC-IV RCS models included its Model 3 covariates. Likelihood ratio tests evaluated the overall association and nonlinear component separately.

In Zigong, receiver operating characteristic (ROC) curves and areas under the curve (AUCs) were calculated for NPNR, PNI, NLR, and SII at 28 and 90 days, with paired comparisons using DeLong tests (27). Incremental prognostic information was examined by comparing a base clinical model with the same model plus log2(NPNR), using Harrell’s C-index, fixed-horizon AUC, and likelihood ratio tests. Optimism-corrected discrimination estimates and differences were obtained from 1,000 bootstrap resamples. Given the small number of deaths, these analyses were interpreted as exploratory, emphasizing effect magnitude and uncertainty rather than statistical significance alone (28).

The primary Zigong analyses used complete cases for missing covariates. Sensitivity analyses included further adjustment for systolic blood pressure and hemoglobin, exclusion of liver disease or malignancy, adjustment for diuretic use, multiple imputation with 20 datasets, ridge-penalized Cox regression, and fixed-horizon logistic regression for 180-day mortality. Multiple imputation used mice (29), and ridge regression used glmnet (30). Core, Firth, and ordered-age analyses retained the same outcome-specific complete-case samples as the original adjusted Cox models. Tertile, spline, subgroup, and incremental discrimination results were interpreted as exploratory because of the limited event counts. Exploratory subgroup interactions considered age, sex, diabetes mellitus, chronic kidney disease, and NYHA class, with Benjamini-Hochberg false discovery rate adjustment. Analyses were performed using R version 4.5.1, with two-sided *p* < 0.05 as a conventional reference threshold alongside effect magnitude, confidence intervals, and consistency across analyses.

## Results

### Zigong discovery cohort

Of the original 2,008 Zigong patients, 1,885 (93.9%) had the laboratory data required for NPNR calculation (Figure 1). Tertile sizes were 629, 628, and 628 patients. The 28-day mortality rates were 0.48%, 1.11%, and 3.50% across T1, T2, and T3; the corresponding fixed-horizon 90-day rates were 0.48%, 1.59%, and 3.82%. Absolute death counts were 3, 7, and 22 at 28 days and 3, 10, and 24 at 90 days. Of the 24 deaths in T3 by 90 days, 23 had a known event time for survival analysis (Supplementary Table S9).

Table 1 summarizes baseline characteristics across NPNR tertiles. Higher NPNR was generally accompanied by higher heart and respiratory rates, a greater proportion of NYHA class IV, higher white blood cell and neutrophil counts, lower lymphocyte counts and albumin, and higher NLR and SII. Urea, creatinine, and BNP tended to be higher, whereas eGFR and sodium tended to be lower. Differences involving the components of NPNR should be interpreted cautiously because some of the separation across tertiles follows directly from the mathematical definition of the index.

**Table 1:** Baseline characteristics of the Zigong discovery cohort according to NPNR tertiles.

Variable	Overall (*n* = 1,885)	T1 (*n* = 629)	T2 (*n* = 628)	T3 (*n* = 628)	*p*-value	SMD
Age					0.396	0.049
≤79	1,180 (62.6)	403 (64.1)	397 (63.2)	380 (60.5)		
>79	705 (37.4)	226 (35.9)	231 (36.8)	248 (39.5)		
Sex					0.035	0.096
Female	1,098 (58.2)	386 (61.4)	371 (59.1)	341 (54.3)		
Male	787 (41.8)	243 (38.6)	257 (40.9)	287 (45.7)		
Weight	52.47 (10.86)	52.22 (10.58)	52.90 (11.06)	52.29 (10.94)	0.478	0.041
BMI	20.78 [18.49, 23.44]	20.70 [18.59, 23.47]	21.20 [18.67, 23.56]	20.55 [18.37, 23.44]	0.062	0.049
Heart rate	85.25 (21.47)	80.33 (19.09)	86.05 (21.28)	89.39 (22.92)	<0.001	0.288
Respiratory rate	19.10 (1.75)	18.86 (1.49)	18.94 (1.37)	19.49 (2.22)	<0.001	0.229
Systolic blood pressure	131.48 (24.63)	130.36 (22.30)	132.62 (24.22)	131.46 (27.12)	0.268	0.062
Diastolic blood pressure	76.66 (14.42)	76.25 (13.31)	77.87 (14.43)	75.86 (15.39)	0.032	0.093
Heart failure type					0.01	0.135
Both	1,399 (74.2)	493 (78.4)	459 (73.1)	447 (71.2)		
Left	437 (23.2)	116 (18.4)	156 (24.8)	165 (26.3)		
Right	49 (2.6)	20 (3.2)	13 (2.1)	16 (2.5)		
NYHA class					0.004	0.147
II	326 (17.3)	123 (19.6)	105 (16.7)	98 (15.6)		
III	979 (51.9)	341 (54.2)	334 (53.2)	304 (48.4)		
IV	580 (30.8)	165 (26.2)	189 (30.1)	226 (36.0)		
Charlson Comorbidity Index	2.00 [1.00, 2.00]	2.00 [1.00, 2.00]	2.00 [1.00, 2.00]	2.00 [1.00, 3.00]	<0.001	0.223
Glasgow Coma Scale	15.00 [15.00, 15.00]	15.00 [15.00, 15.00]	15.00 [15.00, 15.00]	15.00 [15.00, 15.00]	<0.001	0.129
Diabetes mellitus					<0.001	0.243
No	1,448 (76.8)	534 (84.9)	475 (75.6)	439 (69.9)		
Yes	437 (23.2)	95 (15.1)	153 (24.4)	189 (30.1)		
Myocardial infarction					0.003	0.127
No	1748 (92.7)	598 (95.1)	584 (93.0)	566 (90.1)		
Yes	137 (7.3)	31 (4.9)	44 (7.0)	62 (9.9)		
Chronic pulmonary disease					0.039	0.091
No	1,661 (88.1)	571 (90.8)	547 (87.1)	543 (86.5)		
Yes	224 (11.9)	58 (9.2)	81 (12.9)	85 (13.5)		
Chronic kidney disease					0.353	0.085
No	1,438 (76.3)	486 (77.3)	489 (77.9)	463 (73.7)		
Yes	445 (23.6)	143 (22.7)	138 (22.0)	164 (26.1)		
Missing	2 (0.1)	0 (0.0)	1 (0.2)	1 (0.2)		
Liver disease					0.437	0.062
No	1804 (95.7)	604 (96.0)	605 (96.3)	595 (94.7)		
Yes	80 (4.2)	25 (4.0)	23 (3.7)	32 (5.1)		
Missing	1 (0.1)	0 (0.0)	0 (0.0)	1 (0.2)		
Malignancy					0.026	0.1
No	1849 (98.1)	618 (98.3)	622 (99.0)	609 (97.0)		
Yes	36 (1.9)	11 (1.7)	6 (1.0)	19 (3.0)		
White blood cell count	6.50 [5.09, 8.68]	4.70 [3.92, 5.48]	6.47 [5.63, 7.27]	9.77 [8.11, 11.83]	<0.001	1.601
Hemoglobin	114.84 (24.27)	112.59 (24.31)	116.78 (24.06)	115.14 (24.28)	0.009	0.115
Platelet count	135.00 [100.00, 176.00]	116.00 [87.00, 149.00]	141.00 [107.00, 177.00]	152.00 [115.00, 198.00]	<0.001	0.455
Neutrophil count	4.83 [3.61, 6.83]	3.15 [2.57, 3.72]	4.84 [4.33, 5.47]	8.01 [6.72, 10.06]	<0.001	1.972
Lymphocyte count	0.93 [0.62, 1.28]	1.03 [0.71, 1.33]	0.94 [0.64, 1.31]	0.84 [0.52, 1.21]	<0.001	0.158
Albumin	36.51 (4.98)	37.70 (4.32)	37.02 (4.82)	34.82 (5.28)	<0.001	0.394
PNI	41.60 [37.80, 45.45]	43.10 [39.80, 46.75]	42.10 [38.50, 45.60]	39.55 [35.29, 43.50]	<0.001	0.399
NPNR	0.12 [0.09, 0.17]	0.07 [0.06, 0.09]	0.12 [0.10, 0.13]	0.20 [0.17, 0.26]	<0.001	2.254
NLR	5.20 [3.25, 8.75]	3.01 [2.17, 4.26]	5.17 [3.79, 7.33]	10.13 [6.51, 16.70]	<0.001	0.958
SII	709.21 [406.95, 1301.18]	359.56 [246.00, 546.71]	712.05 [502.84, 1033.92]	1526.24 [942.19, 2566.24]	<0.001	0.991
Urea	8.07 [5.91, 11.56]	7.23 [5.51, 10.41]	8.10 [5.93, 11.39]	9.07 [6.55, 13.18]	<0.001	0.284
Creatinine	87.40 [65.00, 123.80]	81.70 [62.40, 112.30]	87.00 [65.35, 122.60]	94.90 [70.00, 139.50]	<0.001	0.178
eGFR	64.27 [41.02, 89.54]	69.83 [45.70, 94.33]	65.38 [41.58, 89.01]	57.40 [36.27, 83.20]	<0.001	0.173
Sodium	138.29 (4.91)	139.32 (4.08)	138.72 (4.59)	136.84 (5.59)	<0.001	0.338
Potassium	3.98 (0.71)	3.92 (0.56)	4.02 (0.72)	4.00 (0.82)	0.023	0.102
Chloride	101.87 (6.02)	103.06 (5.27)	102.39 (5.57)	100.17 (6.74)	<0.001	0.321
Alkaline phosphatase	80.00 [64.00, 102.00]	75.00 [62.00, 96.00]	81.00 [66.00, 100.00]	85.00 [66.00, 110.00]	<0.001	0.176
Total bilirubin	18.30 [12.20, 27.90]	18.80 [12.70, 29.40]	18.65 [12.67, 27.30]	17.40 [11.40, 27.92]	0.081	0.062
BNP	770.76 [324.34, 1767.08]	610.81 [244.64, 1316.80]	812.33 [338.28, 1848.39]	981.21 [403.90, 1999.71]	<0.001	0.217
LVEF	51.00 [41.00, 61.00]	51.00 [41.00, 63.00]	51.00 [41.75, 60.00]	52.00 [41.00, 61.00]	0.678	0.062
RAAS inhibitor					0.042	0.115
No	1,158 (61.4)	374 (59.5)	370 (58.9)	414 (65.9)		
Yes	726 (38.5)	255 (40.5)	257 (40.9)	214 (34.1)		
Missing	1 (0.1)	0 (0.0)	1 (0.2)	0 (0.0)		
Beta-blocker					0.016	0.124
No	1,178 (62.5)	400 (63.6)	362 (57.6)	416 (66.2)		
Yes	706 (37.5)	229 (36.4)	265 (42.2)	212 (33.8)		
Missing	1 (0.1)	0 (0.0)	1 (0.2)	0 (0.0)		
Diuretic					0.641	0.048
No	28 (1.5)	8 (1.3)	11 (1.8)	9 (1.4)		
Yes	1856 (98.5)	621 (98.7)	616 (98.1)	619 (98.6)		
Missing	1 (0.1)	0 (0.0)	1 (0.2)	0 (0.0)		
Nitrate					<0.001	0.202
No	1,434 (76.1)	512 (81.4)	489 (77.9)	433 (68.9)		
Yes	450 (23.9)	117 (18.6)	138 (22.0)	195 (31.1)		
Missing	1 (0.1)	0 (0.0)	1 (0.2)	0 (0.0)		
Digoxin					0.007	0.14
No	946 (50.2)	312 (49.6)	286 (45.5)	348 (55.4)		
Yes	938 (49.8)	317 (50.4)	341 (54.3)	280 (44.6)		
Missing	1 (0.1)	0 (0.0)	1 (0.2)	0 (0.0)		
Vasoactive agents					0.004	0.145
No	1,068 (56.7)	383 (60.9)	365 (58.1)	320 (51.0)		
Yes	816 (43.3)	246 (39.1)	262 (41.7)	308 (49.0)		
Missing	1 (0.1)	0 (0.0)	1 (0.2)	0 (0.0)		

Values are presented as mean (SD), median [IQR], or *n* (%), as appropriate. Category levels are displayed beneath the corresponding variable. SMD, standardized mean difference.

The complete-case Cox analyses included 1,811 patients with 32 deaths at 28 days and 1,810 patients with 36 deaths at 90 days. In the crude 28-day model, each doubling of NPNR was associated with higher mortality (HR 2.429, 95% CI 1.628–3.624, *p* < 0.001). Associations attenuated after adjustment but remained statistically significant in Model 3 at 28 days (HR 1.728, 95% CI 1.130–2.642, *p* = 0.012) and 90 days (HR 1.684, 95% CI 1.124–2.524, *p* = 0.012) (Table 2). Model 1 estimated three coefficients, corresponding to 10.67 and 12.00 events per parameter at 28 and 90 days, whereas Model 3 estimated seven coefficients, corresponding to 4.57 and 5.14 events per parameter (Supplementary Table S6). In the four-parameter core model, the corresponding HRs were 2.163 (95% CI 1.444–3.240) and 2.115 (95% CI 1.442–3.102). Firth-penalized core-model HRs were 2.170 (95% CI 1.442–3.233) and 2.122 (95% CI 1.440–3.096), respectively (Supplementary Table S7 and Supplementary Figure S3).

**Table 2:** Association of NPNR with 28-day and 90-day all-cause mortality in the Zigong discovery cohort.

Variable	Crude		Model 1		Model 2		Model 3	
	HR (95% CI)	*P*-value	HR (95% CI)	*P*-value	HR (95% CI)	*P*-value	HR (95% CI)	*P*-value
28-day all-cause mortality								
NPNR per doubling	2.429 (1.628–3.624)	<0.001	2.466 (1.637–3.715)	<0.001	2.067 (1.366–3.127)	<0.001	1.728 (1.130–2.642)	0.012
NPNR tertiles								
T1	1.00 (Ref)		1.00 (Ref)		1.00 (Ref)		1.00 (Ref)	
T2	2.343 (0.606–9.062)	0.217	2.334 (0.604–9.028)	0.219	2.104 (0.543–8.158)	0.282	1.819 (0.468–7.070)	0.388
T3	7.513 (2.249–25.102)	0.001	7.357 (2.200–24.611)	0.001	5.822 (1.719–19.722)	0.005	4.483 (1.304–15.408)	0.017
*P* for trend		<0.001		<0.001		<0.001		0.004
90-day all-cause mortality								
NPNR per doubling	2.353 (1.611–3.437)	<0.001	2.395 (1.625–3.530)	<0.001	1.996 (1.347–2.959)	<0.001	1.684 (1.124–2.524)	0.012
NPNR tertiles								
T1	1.00 (Ref)		1.00 (Ref)		1.00 (Ref)		1.00 (Ref)	
T2	3.349 (0.922–12.168)	0.066	3.334 (0.918–12.116)	0.067	2.986 (0.820–10.877)	0.097	2.580 (0.707–9.424)	0.151
T3	7.882 (2.367–26.252)	<0.001	7.731 (2.319–25.775)	<0.001	5.986 (1.774–20.205)	0.004	4.581 (1.339–15.670)	0.015
*P* for trend		<0.001		<0.001		0.001		0.008

Crude: unadjusted. Model 1: adjusted for age (>79 vs. ≤79 years) and sex. Model 2: additionally adjusted for CCI and stratified by NYHA class. Model 3: additionally adjusted for standardized ln(BNP), standardized eGFR, and standardized sodium, with continued stratification by NYHA class. Continuous NPNR is expressed per doubling.

An increasing mortality gradient was observed across NPNR tertiles. In Model 3, the HRs for T2 and T3 versus T1 were 1.819 (95% CI 0.468–7.070, *p* = 0.388) and 4.483 (95% CI 1.304–15.408, *p* = 0.017), respectively, at 28 days, and 2.580 (95% CI 0.707–9.424, *p* = 0.151) and 4.581 (95% CI 1.339–15.670, *p* = 0.015) at 90 days. *P* for trend was 0.004 and 0.008, respectively. As T1 contained only three deaths at each time horizon, the wide confidence intervals and relatively large tertile HRs require caution; continuous NPNR remained the primary effect measure.

Kaplan–Meier curves showed clear separation in 28-day and 90-day survival probabilities across NPNR tertiles, with the highest NPNR group having the lowest survival probability; log-rank *p* values were <0.001 for both outcomes (Figure 2). The numbers at risk remained relatively stable among patients without events during the 28-day and 90-day follow-up periods, consistent with the fixed short-term follow-up structure of the Zigong database.

**Figure 2:**
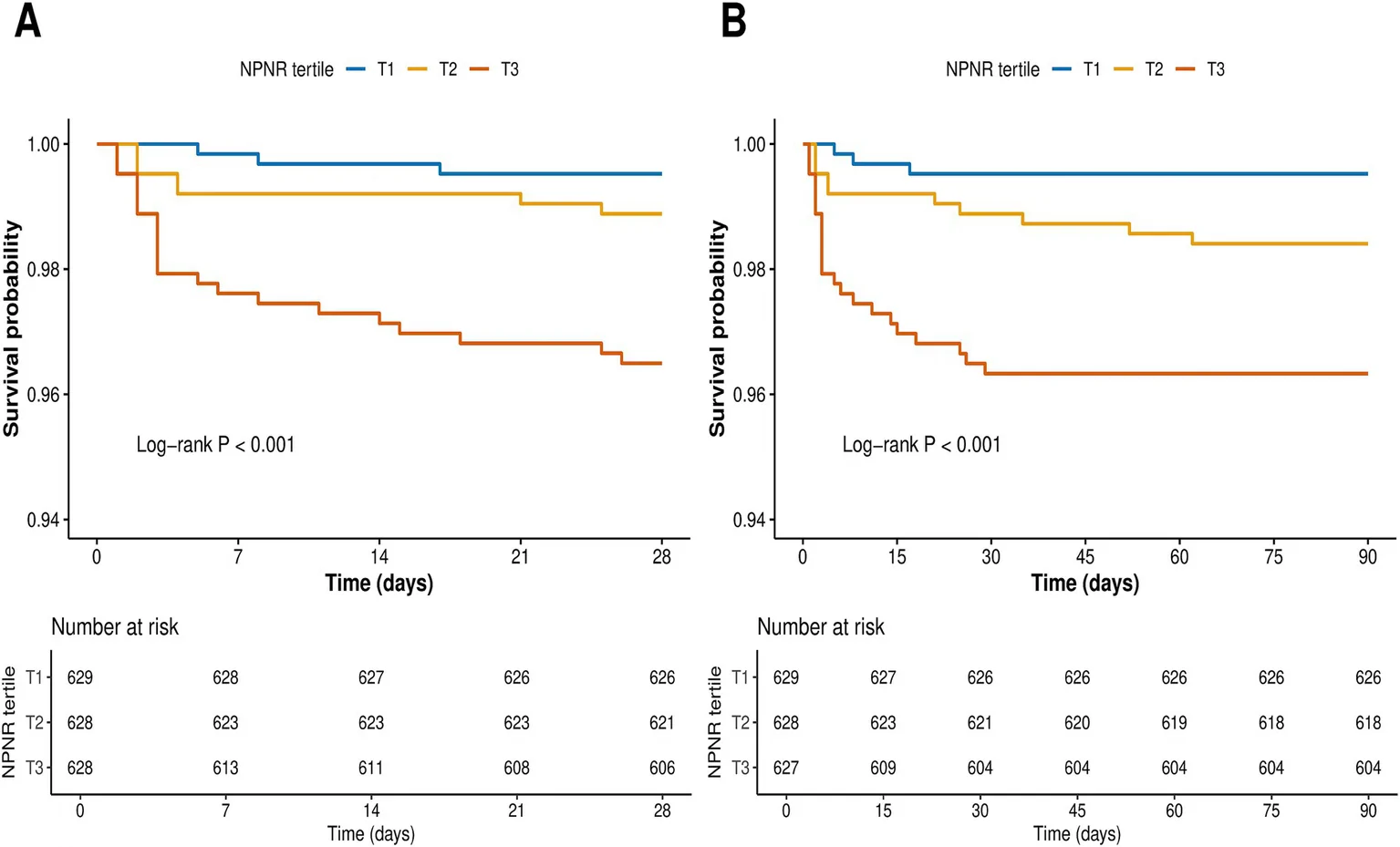
Kaplan–Meier survival curves according to NPNR tertiles in the Zigong discovery cohort. **(A)** 28-day all-cause mortality. **(B)** 90-day all-cause mortality. Numbers at risk are shown below each curve; log-rank *p* < 0.001 for both time horizons.

In the Zigong RCS analyses, the exposure-response association showed evidence of nonlinearity. The 28-day knots were approximately 0.065, 0.115, and 0.243, with 0.115 as the reference; overall and nonlinearity *p*-values were 0.012 and 0.009, respectively. The corresponding 90-day *p*-values were 0.016 and 0.013 (Figure 3). Risk appeared to rise more rapidly at lower-to-moderate NPNR levels before approaching a plateau, although confidence intervals widened at higher values with few observations and deaths. These findings do not support a fixed clinical cutoff.

**Figure 3:**
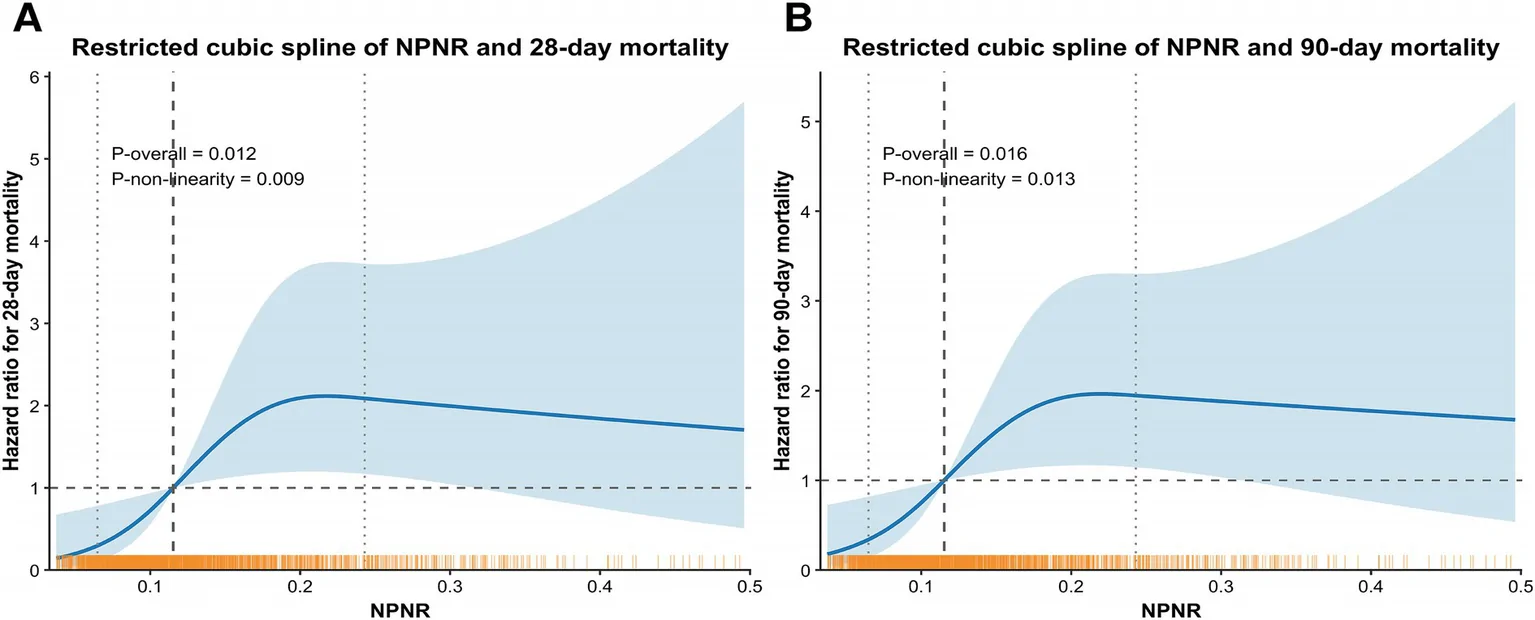
Restricted cubic spline analyses of NPNR and short-term all-cause mortality in the Zigong discovery cohort. **(A)** 28-day mortality. **(B)** 90-day mortality. Three knots were placed at the 10th, 50th, and 90th percentiles, with the median as the reference. Models used the covariate set of Model 3 and were stratified by NYHA class.

At 28 days, the NPNR AUC was 0.727 (95% CI 0.637–0.816); at 90 days, it was 0.717 (95% CI 0.636–0.799) (Figure 4 and Table 3). NPNR did not significantly outperform PNI, NLR, or SII in DeLong comparisons at either horizon (28-day *p* = 0.136, 0.472, and 0.941; 90-day *p* = 0.091, 0.287, and 0.793, respectively). Thus, although stand-alone discrimination was moderate, the data did not demonstrate superiority over established comparator indices.

**Figure 4:**
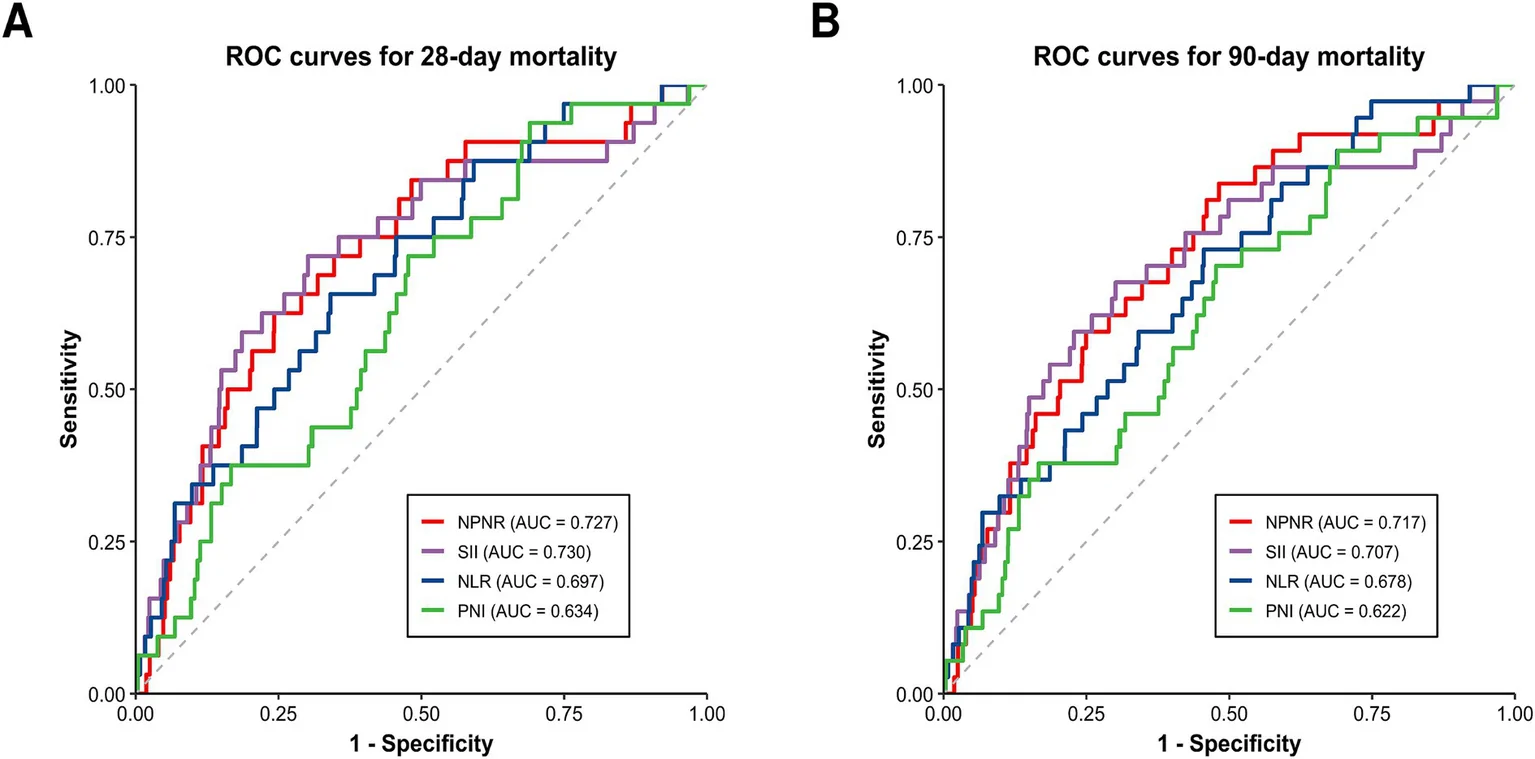
Receiver operating characteristic curves for NPNR, PNI, NLR, and SII in the Zigong discovery cohort. **(A)** 28-day all-cause mortality. **(B)** 90-day all-cause mortality. DeLong tests did not demonstrate significant superiority of NPNR over PNI, NLR, or SII.

**Table 3:** Discrimination and incremental prognostic information in the Zigong discovery cohort.

Marker	28-day mortality			90-day mortality		
	AUC (95% CI)	Sensitivity	Specificity	AUC (95% CI)	Sensitivity	Specificity
NPNR	0.727 (0.637–0.816)	62.50%	75.80%	0.717 (0.636–0.799)	83.80%	51.80%
PNI	0.634 (0.544–0.723)	93.80%	31.00%	0.622 (0.532–0.713)	70.30%	52.30%
NLR	0.697 (0.608–0.787)	65.60%	65.90%	0.678 (0.594–0.762)	73.00%	54.50%
SII	0.730 (0.631–0.828)	71.90%	69.90%	0.707 (0.614–0.801)	67.60%	69.90%
Incremental prognostic information						
Outcome	Base C-index, corrected	Base + NPNR C-index, corrected	Corrected ΔC-index	LRT P-value	Corrected ΔAUC	DeLong P-value
28-day	0.717 (0.629–0.803)	0.738 (0.662–0.809)	0.022 (−0.030–0.043)	0.013	0.024 (−0.010–0.039)	0.089
90-day	0.708 (0.626–0.789)	0.735 (0.658–0.808)	0.027 (−0.013–0.045)	0.013	0.024 (−0.011–0.041)	0.081

AUC, area under the receiver operating characteristic curve. Incremental discrimination estimates were optimism-corrected using 1,000 bootstrap resamples. These analyses are interpreted as exploratory incremental prognostic information rather than validation of a stand-alone prediction model.

Incremental discrimination was also limited. For 28-day mortality, the apparent base-model C-index increased from 0.753 to 0.781 after NPNR was added, while the optimism-corrected C-index rose from 0.717 to 0.738 (corrected ΔC-index 0.022; 95% interval crossed zero). At 90 days, the corrected C-index increased from 0.708 to 0.735 (corrected ΔC-index 0.027; interval crossed zero). Likelihood ratio test *p* values were 0.013 at both horizons, but corrected ΔAUC intervals also included zero (Table 3). These exploratory results do not establish a consistent or clinically meaningful improvement in predictive performance.

### Sensitivity analyses

The association remained directionally consistent across several sensitivity analyses. Additional adjustment for systolic blood pressure and hemoglobin yielded HRs of 1.792 (95% CI 1.170–2.744, *p* = 0.007) at 28 days and 1.744 (95% CI 1.163–2.615, *p* = 0.007) at 90 days. Excluding liver disease or malignancy yielded HRs of 1.798 and 1.692, and further adjustment for diuretic use yielded HRs of 1.716 and 1.672. Multiple imputation gave HRs of 1.730 (95% CI 1.105–2.707, *p* = 0.019) and 1.687 (95% CI 1.105–2.578, *p* = 0.017). Ridge-penalized estimates remained above 1, and fixed-horizon 180-day analysis yielded an OR of 1.596 (95% CI 1.103–2.304, *p* = 0.013) per doubling of NPNR (Supplementary Table S3). With the original eight age categories represented as an ordered score, the Model 3 HRs were 1.725 (95% CI 1.126–2.640, *p* = 0.012) and 1.679 (95% CI 1.119–2.520, *p* = 0.012) at 28 and 90 days (Supplementary Table S8).

The proportional hazards assessment showed no evidence that log2(NPNR) itself violated the PH assumption (*p* = 0.485 for 28-day mortality and *p* = 0.391 for 90-day mortality). The corresponding global test *p* values were 0.074 and 0.259. In the 28-day model, the scaled Schoenfeld residual test for standardized serum sodium yielded *p* = 0.029, suggesting a possible time-varying effect for this covariate; however, neither the global model nor the primary exposure showed clear evidence of PH violation (Supplementary Table S2). In exploratory subgroup analyses, none of the 28-day interaction tests remained below 0.05 after FDR correction. For 90-day mortality, the interaction with diabetes had an FDR-adjusted *q*-value of 0.036. Given the limited number of events and the lack of consistent interaction patterns across other subgroups, this finding was considered hypothesis-generating and was not incorporated into the main conclusions (Supplementary Figure S1 and Supplementary Table S4).

### MIMIC-IV external replication

The MIMIC-IV replication cohort consisted of 1,150 unique adult ICU patients with HF. The median NPNR was 0.274 (IQR 0.173–0.438), which was notably higher than that in the Zigong cohort. The tertile cut points were 0.206 and 0.371, with 384 patients in T1, 383 in T2, and 383 in T3. The 28-day mortality rates were 21.9%, 29.8%, and 35.2% across T1, T2, and T3, respectively, while the corresponding 90-day mortality rates were 30.2%, 37.1%, and 45.7%. The mean age of the replication cohort was 69.45 ± 14.36 years, 58.9% were male, and the cohort had a greater burden of comorbidities and organ dysfunction than the discovery cohort (Table 4).

**Table 4:** Baseline characteristics of the MIMIC-IV external replication cohort according to NPNR tertiles.

Variable	Overall (*n* = 1,150)	T1 (*n* = 384)	T2 (*n* = 383)	T3 (*n* = 383)	*P*-value
Age	69.45 (14.36)	67.04 (14.79)	70.69 (14.09)	70.63 (13.93)	<0.001
Sex					0.232
Female	473 (41.1)	160 (41.7)	145 (37.9)	168 (43.9)	
Male	677 (58.9)	224 (58.3)	238 (62.1)	215 (56.1)	
BMI	30.05 (8.83)	29.66 (8.93)	29.99 (8.22)	30.49 (9.31)	0.424
Systolic blood pressure	111.18 (14.63)	112.39 (15.90)	112.07 (14.83)	109.09 (12.78)	0.003
Charlson Comorbidity Index	7.00 [5.00, 9.00]	7.00 [5.00, 8.00]	7.00 [5.00, 9.00]	7.00 [5.00, 9.00]	0.152
Glasgow Coma Scale	13.51 (3.02)	13.58 (2.88)	13.40 (3.14)	13.56 (3.03)	0.646
Diabetes mellitus					0.588
No	671 (58.3)	232 (60.4)	218 (56.9)	221 (57.7)	
Yes	479 (41.7)	152 (39.6)	165 (43.1)	162 (42.3)	
Chronic pulmonary disease					0.702
No	784 (68.2)	257 (66.9)	260 (67.9)	267 (69.7)	
Yes	366 (31.8)	127 (33.1)	123 (32.1)	116 (30.3)	
Chronic kidney disease					0.991
No	671 (58.3)	223 (58.1)	224 (58.5)	224 (58.5)	
Yes	479 (41.7)	161 (41.9)	159 (41.5)	159 (41.5)	
Myocardial infarction					0.017
No	751 (65.3)	268 (69.8)	230 (60.1)	253 (66.1)	
Yes	399 (34.7)	116 (30.2)	153 (39.9)	130 (33.9)	
Hemoglobin	10.44 (2.00)	10.38 (1.90)	10.57 (2.12)	10.36 (1.97)	0.277
Platelet count	199.77 (104.45)	174.00 (86.73)	200.79 (99.55)	224.57 (118.64)	<0.001
White blood cell count	12.75 [8.60, 17.90]	7.90 [5.60, 10.70]	12.20 [9.80, 15.50]	18.80 [15.50, 25.90]	<0.001
Albumin	30.06 (5.84)	32.55 (5.58)	30.62 (5.04)	27.02 (5.49)	<0.001
Neutrophil count	10.07 [6.62, 15.21]	5.43 [3.93, 6.81]	10.08 [8.78, 11.92]	17.36 [14.60, 23.07]	<0.001
Lymphocyte count	1.08 [0.66, 1.66]	1.09 [0.64, 1.66]	1.13 [0.71, 1.78]	1.01 [0.61, 1.60]	0.159
Blood urea nitrogen	31.00 [19.00, 51.00]	27.00 [17.75, 47.00]	31.00 [19.00, 49.00]	35.00 [21.00, 54.00]	<0.001
Creatinine	1.60 [1.00, 2.80]	1.50 [1.00, 2.70]	1.60 [1.00, 2.80]	1.90 [1.10, 2.90]	0.016
eGFR	39.68 [21.16, 69.04]	47.65 [22.53, 73.19]	39.68 [21.29, 69.99]	32.28 [20.55, 56.55]	0.002
Sodium	138.88 (5.52)	138.72 (5.29)	139.25 (5.60)	138.68 (5.67)	0.277
Potassium	4.52 (0.79)	4.45 (0.71)	4.50 (0.80)	4.62 (0.83)	0.006
Beta-blocker					0.123
No	386 (33.6)	121 (31.5)	121 (31.6)	144 (37.6)	
Yes	764 (66.4)	263 (68.5)	262 (68.4)	239 (62.4)	
ACEI/ARB					<0.001
No	791 (68.8)	234 (60.9)	260 (67.9)	297 (77.5)	
Yes	359 (31.2)	150 (39.1)	123 (32.1)	86 (22.5)	
Diuretic					0.61
No	256 (22.3)	92 (24.0)	81 (21.1)	83 (21.7)	
Yes	894 (77.7)	292 (76.0)	302 (78.9)	300 (78.3)	

Values are presented as mean (SD), median [IQR], or *n* (%), as appropriate. Category levels are displayed beneath the corresponding variable. NPNR tertile cut points were determined within the MIMIC-IV replication cohort.

In MIMIC-IV, Model 3 yielded HRs per doubling of NPNR of 1.112 (95% CI 1.000–1.237, *p* = 0.049) for 28-day mortality and 1.081 (95% CI 0.986–1.186, *p* = 0.096) for 90-day mortality (Table 5). With continuous standardized age in place of dichotomized age, the corresponding HRs were 1.111 (95% CI 0.999–1.235, *p* = 0.052) and 1.078 (95% CI 0.984–1.182, *p* = 0.107) (Supplementary Table S8). For T3 versus T1, Model 3 HRs were 1.444 (95% CI 1.096–1.904, *p* = 0.009) at 28 days and 1.397 (95% CI 1.101–1.774, *p* = 0.006) at 90 days; *P* for trend was 0.014 and 0.006, respectively.

**Table 5:** Association of NPNR with 28-day and 90-day all-cause mortality in the MIMIC-IV external replication cohort.

Variable	Crude		Model 1		Model 2		Model 3	
	HR (95% CI)	*P*-value	HR (95% CI)	*P*-value	HR (95% CI)	*P*-value	HR (95% CI)	*P*-value
28-day all-cause mortality								
NPNR per doubling	1.189 (1.069–1.323)	0.001	1.171 (1.053–1.303)	0.004	1.140 (1.026–1.266)	0.015	1.112 (1.000–1.237)	0.049
NPNR tertiles								
T1	1.00 (Ref)		1.00 (Ref)		1.00 (Ref)		1.00 (Ref)	
T2	1.469 (1.108–1.948)	0.007	1.384 (1.043–1.836)	0.025	1.367 (1.030–1.814)	0.03	1.300 (0.978–1.727)	0.07
T3	1.779 (1.355–2.336)	<0.001	1.667 (1.267–2.194)	<0.001	1.548 (1.176–2.039)	0.002	1.444 (1.096–1.904)	0.009
*P* for trend								0.014
90-day all-cause mortality								
NPNR per doubling	1.155 (1.053–1.267)	0.002	1.137 (1.036–1.248)	0.007	1.104 (1.008–1.209)	0.033	1.081 (0.986–1.186)	0.096
NPNR tertiles								
T1	1.00 (Ref)		1.00 (Ref)		1.00 (Ref)		1.00 (Ref)	
T2	1.332 (1.042–1.702)	0.022	1.254 (0.980–1.604)	0.072	1.240 (0.970–1.587)	0.086	1.194 (0.932–1.529)	0.16
T3	1.716 (1.357–2.171)	<0.001	1.606 (1.267–2.034)	<0.001	1.481 (1.168–1.878)	0.001	1.397 (1.101–1.774)	0.006
*P* for trend								0.006

Crude: unadjusted. Model 1: adjusted for age (>79 vs. ≤79 years) and sex. Model 2: additionally adjusted for CCI and standardized systolic blood pressure. Model 3: additionally adjusted for standardized eGFR and standardized sodium. Continuous NPNR is expressed per doubling.

In MIMIC-IV, Kaplan–Meier curves separated across NPNR tertiles (log-rank *p* < 0.001 at both horizons; Figure 5). RCS overall association *p* values were 0.016 at 28 days and 0.003 at 90 days, without clear evidence of nonlinearity (*p* = 0.841 and 0.491, respectively; Figure 6). The exposure-risk curves were closer to monotonic than in Zigong. These findings indicated a broadly similar risk direction across tertiles, with attenuated continuous associations and a different exposure-response shape.

**Figure 5:**
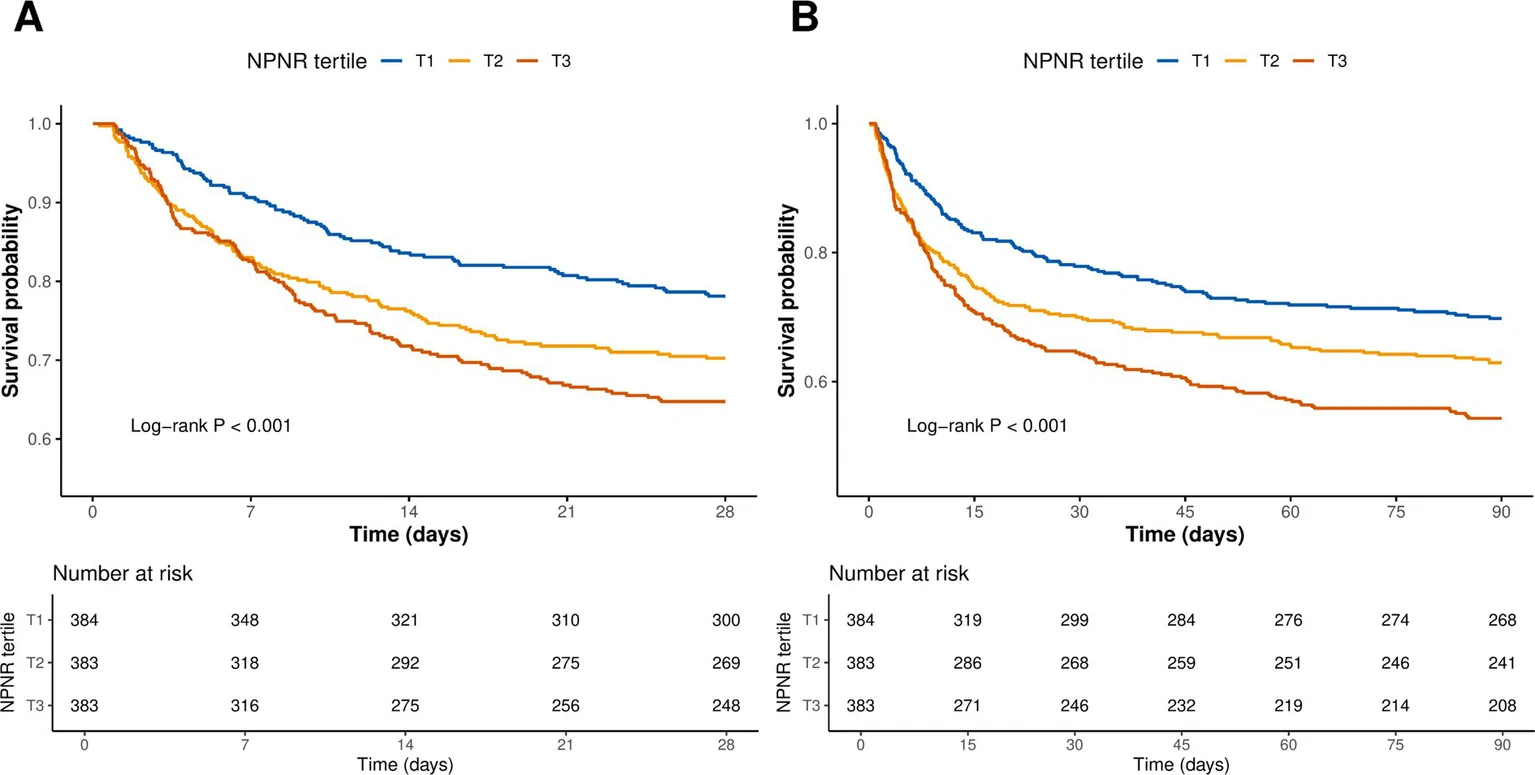
Kaplan–Meier survival curves according to cohort-specific NPNR tertiles in the MIMIC-IV external replication cohort. **(A)** 28-day all-cause mortality. **(B)** 90-day all-cause mortality. Log-rank *p* < 0.001 for both time horizons.

**Figure 6:**
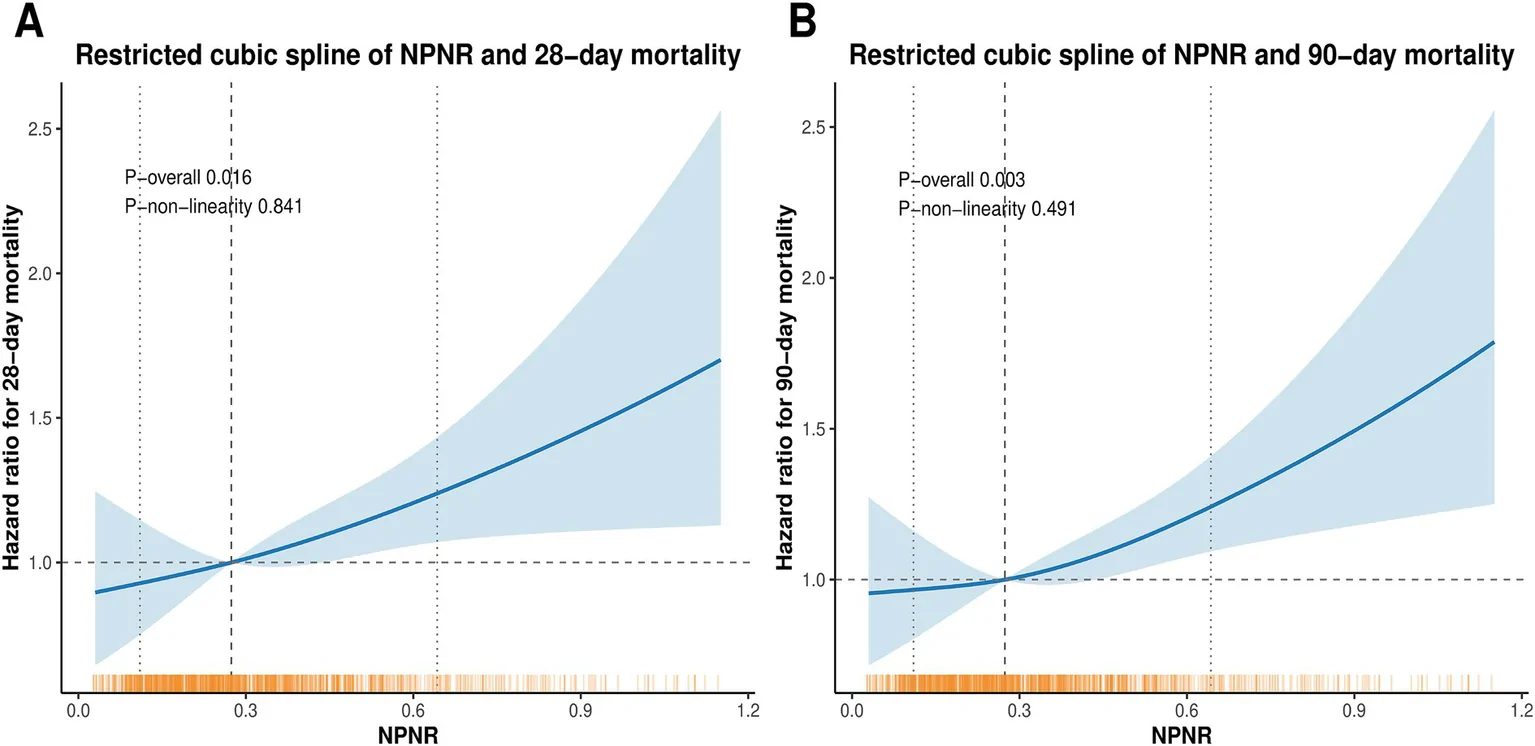
Restricted cubic spline analyses of NPNR and short-term all-cause mortality in the MIMIC-IV external replication cohort. **(A)** 28-day mortality: *P*-overall = 0.016 and *P*-nonlinearity = 0.841. **(B)** 90-day mortality: *P*-overall = 0.003 and *P*-non-linearity = 0.491.

## Discussion

The main finding was an association between higher NPNR and short-term all-cause mortality in the Zigong HF cohort. Effect estimates remained directionally similar in parsimonious and Firth-penalized Cox models, although only 32 and 36 deaths were available for the respective survival analyses. Stand-alone discrimination was moderate, without significant superiority over PNI, NLR, or SII or a reliable improvement when NPNR was added to a clinical model. In MIMIC-IV, continuous associations were attenuated, particularly at 90 days. Taken together, the results suggest a possible adjunctive marker rather than an externally validated prediction tool.

The PNI combines albumin and lymphocyte count and has previously been associated with HF outcomes (15, 16). In hospitalized patients with HF, albumin is affected by the acute-phase response, hepatic synthesis, renal dysfunction, congestion, and fluid shifts (17, 18), limiting its specificity for any single underlying process. Validated nutritional assessment data were unavailable, precluding an assessment of whether NPNR reflects nutritional status. NPNR is therefore more appropriately regarded as an exploratory laboratory composite associated with systemic inflammation and overall illness severity rather than as a nutritional marker.

The biological interpretation of NPNR also requires attention to its components. The index is derived from neutrophil count, lymphocyte count, and albumin, creating mathematical overlap with NLR, PNI, and SII. Differences in these measurements across NPNR tertiles consequently do not establish a distinct biological pathway. Although neutrophil-related inflammation is relevant to HF (6–12), the independent contribution of neutrophils cannot be separated reliably from albumin, lymphocytes, disease severity, and residual confounding in the present observational data.

Previous studies have linked NLR, SII, and PNI with HF outcomes (13–16). A recent HFpEF study examined the neutrophil percentage-to-albumin ratio as an inflammatory index associated with diuretic resistance and mortality (19), providing context for combined neutrophil-albumin indices. Nevertheless, NPNR showed no significant DeLong advantage over PNI, NLR, or SII, and bootstrap-corrected C-index changes were small with intervals crossing zero. Its incremental prognostic contribution therefore remains uncertain, and the findings do not justify replacing established indices.

Li et al. previously reported an association between NPNR and long-term mortality after acute myocardial infarction (20). The present HF analysis addresses shorter follow-up intervals but yielded different exposure-response patterns: the Zigong RCS suggested nonlinearity, while the MIMIC-IV curve was approximately monotonic. Differences in baseline risk, ICU acuity, sampling timing, albumin distribution, and residual confounding may contribute to this contrast. Sparse deaths in Zigong also limit the precision of spline-shape inference. Accordingly, a universal NPNR threshold is not supported, and continuous interpretation is preferable.

Clinical severity differed substantially between the two cohorts. Ninety-day mortality was approximately 2.0% in Zigong and 37.7% in MIMIC-IV, with median NPNR values of approximately 0.115 and 0.274, respectively. Differences in underlying risk, comorbidity burden, clinical setting, and laboratory profiles may explain part of the smaller continuous HRs in MIMIC-IV. The ICU cohort therefore provides an assessment of association direction in a higher-acuity setting, not formal validation of an NPNR-based prediction model.

The attenuated continuous estimates in MIMIC-IV reinforce this distinction: the 28-day association was borderline significant (*p* = 0.049), whereas the 90-day association did not reach conventional significance (*p* = 0.096). With continuous age adjustment, the corresponding *p*-values were 0.052 and 0.107. Despite the tertile gradients, Kaplan–Meier separation, and significant overall RCS tests, identical effect sizes and exposure-response shapes were not demonstrated across cohorts. We therefore interpret the MIMIC-IV findings as partial external replication rather than formal external validation.

The NPNR can be calculated from routinely available laboratory tests and may serve as an exploratory adjunct to comprehensive HF assessment. Higher values may coincide with greater inflammatory or illness-related burden, but their interpretation should remain secondary to established HF assessments and validated prognostic tools. However, the present data do not establish reliable improvement in predictive performance; NPNR should not be used independently for treatment selection or routine decision thresholds.

Strengths of this study include the use of two independent cohorts with distinct clinical settings, emphasis on continuous NPNR, and transparent reporting of both positive associations and neutral discrimination comparisons. Progressively adjusted models, a simpler core model, Firth and ridge penalization, multiple imputation, and other sensitivity analyses provided complementary assessments of estimate stability. None of these methods, however, eliminates uncertainty arising from the limited number of deaths.

Several limitations warrant emphasis. First, only 32 and 36 deaths with known event times occurred at 28 and 90 days in Zigong. Model 3 estimated seven coefficients, corresponding to 4.57 and 5.14 events per parameter; overfitting remains possible, and the large tertile HRs, spline shapes, subgroup findings, and incremental prediction analyses remain exploratory. Second, LVEF was unavailable for 1,281 patients (67.96%) in Zigong, precluding reliable HFrEF, HFmrEF, or HFpEF classification and limiting phenotype-specific interpretation and generalizability. Third, residual confounding and reverse causation cannot be excluded in this retrospective study. Serial inflammatory measurements were unavailable, and the absence of validated nutritional assessments prevents interpreting NPNR as a nutritional indicator. Finally, the greater acuity and different care environment in MIMIC-IV limit direct comparisons between cohorts. Future prospective work should include more complete HF phenotyping, standardized inflammatory measurements, and evaluation of incremental prognostic value against existing models.

## Conclusion

In conclusion, we observed an association between higher NPNR and 28-day and 90-day all-cause mortality in the Zigong HF cohort, with attenuated and incomplete replication in MIMIC-IV. Limited death events and extensive LVEF missingness constrain the certainty and phenotype-specific applicability of these findings. NPNR may be viewed as an exploratory laboratory index associated with inflammation and overall illness severity, rather than a validated stand-alone prediction tool. It did not demonstrate superior discrimination over PNI, NLR, or SII, and its incremental prognostic value remains uncertain.

## Data Availability

The Zigong heart failure dataset and MIMIC-IV are available through PhysioNet under their respective data-use requirements. MIMIC-IV access requires completion of the applicable training, credentialing, and data-use agreement. Analysis definitions and reproducible code may be shared in accordance with journal policy and the licensing requirements of the source databases.
