# Supplementary material for: Neutrophil-to-prognostic nutritional index ratio and short-term mortality in heart failure: an exploratory association without demonstrated superiority over PNI, NLR, or SII
Source: Front Nutr. 2026 Oct 23;13:1995940. doi: 10.3389/fnut.2026.1995940 (PMC13739510; doi:10.3389/fnut.2026.1995940)
Supplement: Supplementary file 1 [file Supplementary_file_1.DOCX]

**Supplementary Material**

**Supplementary Table S1. Missingness of NPNR components and selected covariates in the Zigong cohort**

| **Variable** | **Missing, n** | **Missing, %** |
| --- | --- | --- |
| Neutrophil count | 27 | 1.34 |
| Lymphocyte count | 27 | 1.34 |
| Albumin | 102 | 5.08 |
| Any NPNR component | 123 | 6.13 |
| LVEF | 1281 | 67.96 |
| eGFR | 46 | 2.44 |
| BNP | 22 | 1.17 |
| Urea | 8 | 0.42 |
| Creatinine | 8 | 0.42 |
| Charlson Comorbidity Index | 5 | 0.27 |
| Sodium | 3 | 0.16 |

**Supplementary Table S2. Proportional hazards assumption for the primary Cox models in the Zigong cohort**

| **Variable** | **28-day mortality** | | | **90-day mortality** | | |
| --- | --- | --- | --- | --- | --- | --- |
|  |  |  |  |  |  |  |
|  |  |  |  |  |  |  |
|  | **Chi-square** | **df** | **P-value** | **Chi-square** | **df** | **P-value** |
| log2(NPNR) | 0.488 | 1 | 0.485 | 0.735 | 1 | 0.391 |
| Standardized sodium | 4.758 | 1 | 0.029 | 3.607 | 1 | 0.058 |
| Global test | 12.931 | 7 | 0.074 | 8.919 | 7 | 0.259 |

**Supplementary Table S3. Sensitivity analyses in the Zigong cohort**

| **Analysis** | **28-day mortality** | | | **90-day mortality** | | |
| --- | --- | --- | --- | --- | --- | --- |
|  |  |  |  |  |  |  |
|  |  |  |  |  |  |  |
|  | **N / events** | **HR (95% CI)** | **P-value** | **N / events** | **HR (95% CI)** | **P-value** |
| Model 3 + systolic blood pressure + hemoglobin | 1810 / 32 | 1.792 (1.170–2.744) | 0.007 | 1809 / 36 | 1.744 (1.163–2.615) | 0.007 |
| Excluding liver disease or malignancy | 1702 / 26 | 1.798 (1.132–2.855) | 0.013 | 1701 / 28 | 1.692 (1.077–2.659) | 0.023 |
| Model 3 + diuretic | 1810 / 32 | 1.716 (1.120–2.630) | 0.013 | 1809 / 36 | 1.672 (1.114–2.510) | 0.013 |
| Multiple imputation (m = 20) | 1885 / 32 | 1.730 (1.105–2.707) | 0.019 | 1884 / 36 | 1.687 (1.105–2.578) | 0.017 |
|  |  |  |  |  |  |  |
| **180-day fixed-horizon sensitivity** |  |  |  |  |  |  |
| **Analysis** | **N / events** | **OR (95% CI)** | **P-value** | **Exposure scale** |  |  |
| 180-day fixed-horizon logistic regression | 1811 / 51 | 1.596 (1.103–2.304) | 0.013 | NPNR per doubling |  |  |

Cox sensitivity analyses report HRs for each doubling of NPNR. The 180-day fixed-horizon analysis reports an OR for each doubling of NPNR.

**Supplementary Table S4. Exploratory subgroup interaction tests in the Zigong cohort**

| **Subgroup** | **28-day mortality** | | **90-day mortality** | |
| --- | --- | --- | --- | --- |
|  |  |  |  |  |
|  | **P for interaction** | **FDR q** | **P for interaction** | **FDR q** |
| Age | 0.082 | 0.137 | 0.247 | 0.412 |
| Sex | 0.827 | 0.911 | 0.845 | 0.955 |
| Diabetes mellitus | 0.028 | 0.07 | 0.007 | 0.036 |
| Chronic kidney disease | 0.022 | 0.07 | 0.063 | 0.157 |
| NYHA class | 0.911 | 0.911 | 0.955 | 0.955 |

Interaction P-values were adjusted using the Benjamini-Hochberg false discovery rate procedure. Subgroup analyses were exploratory.

**Supplementary Table S5. DeLong comparisons of NPNR with PNI, NLR, and SII in the Zigong cohort**

| **Comparison** | **28-day mortality** | | | **90-day mortality** | | |
| --- | --- | --- | --- | --- | --- | --- |
|  |  |  |  |  |  |  |
|  |  |  |  |  |  |  |
|  | **NPNR AUC** | **Comparator AUC** | **P-value** | **NPNR AUC** | **Comparator AUC** | **P-value** |
| NPNR vs PNI | 0.727 | 0.634 | 0.136 | 0.717 | 0.622 | 0.091 |
| NPNR vs NLR | 0.727 | 0.697 | 0.472 | 0.717 | 0.678 | 0.287 |
| NPNR vs SII | 0.727 | 0.73 | 0.941 | 0.717 | 0.707 | 0.793 |

**Supplementary Table S6. Model degrees of freedom and events per parameter in the Zigong cohort**

| **Outcome** | **Model** | **N** | **Deaths** | **df** | **Events/df** |
| --- | --- | --- | --- | --- | --- |
| 28-day | Model 1 | 1811 | 32 | 3 | 10.67 |
| 28-day | Core model | 1811 | 32 | 4 | 8.00 |
| 28-day | Model 2 | 1811 | 32 | 4 | 8.00 |
| 28-day | Model 3 | 1811 | 32 | 7 | 4.57 |
| 28-day | Tertile Model 3 | 1811 | 32 | 8 | 4.00 |
| 28-day | 3-knot RCS + Model 3 | 1811 | 32 | 8 | 4.00 |
| 90-day | Model 1 | 1810 | 36 | 3 | 12.00 |
| 90-day | Core model | 1810 | 36 | 4 | 9.00 |
| 90-day | Model 2 | 1810 | 36 | 4 | 9.00 |
| 90-day | Model 3 | 1810 | 36 | 7 | 5.14 |
| 90-day | Tertile Model 3 | 1810 | 36 | 8 | 4.50 |
| 90-day | 3-knot RCS + Model 3 | 1810 | 36 | 8 | 4.50 |

Events/df is descriptive, not a guarantee against overfitting. NYHA was a stratification factor in Models 2–3 and the expanded spline models; it adds baseline-hazard strata but no estimated NYHA regression coefficient. The three-knot spline adds two NPNR coefficients. Main analyses used identical complete-case datasets across models.

**Supplementary Table S7. Parsimonious and Firth-penalized Cox sensitivity analyses in the Zigong cohort**

| **Outcome** | **Model** | **N** | **Deaths** | **HR (95% CI)** | **P-value** |
| --- | --- | --- | --- | --- | --- |
| 28-day | Model 1 | 1811 | 32 | 2.466 (1.637–3.715) | <0.001 |
| 28-day | Core model | 1811 | 32 | 2.163 (1.444–3.240) | <0.001 |
| 28-day | Firth Model 1 | 1811 | 32 | 2.476 (1.634–3.706) | <0.001 |
| 28-day | Firth core | 1811 | 32 | 2.170 (1.442–3.233) | <0.001 |
| 90-day | Model 1 | 1810 | 36 | 2.395 (1.625–3.530) | <0.001 |
| 90-day | Core model | 1810 | 36 | 2.115 (1.442–3.102) | <0.001 |
| 90-day | Firth Model 1 | 1810 | 36 | 2.404 (1.622–3.522) | <0.001 |
| 90-day | Firth core | 1810 | 36 | 2.122 (1.440–3.096) | <0.001 |

Effect estimates are per doubling of NPNR. Model 1 adjusted for age category (>79 vs ≤79 years) and sex. The core model adjusted for age category, sex, and NYHA IV versus II/III. Firth models used the same corresponding covariates; their 95% confidence intervals were based on penalized profile likelihood. Expanded Model 3 was not re-fitted using Firth penalization.

**Supplementary Table S8. Alternative age coding sensitivity analyses in both cohorts**

| **Cohort** | **Outcome** | **Age handling** | **N** | **Deaths** | **HR (95% CI)** | **P-value** |
| --- | --- | --- | --- | --- | --- | --- |
| Zigong | 28-day | Original eight-category age as ordinal score | 1811 | 32 | 1.725 (1.126–2.640) | 0.012 |
| MIMIC-IV | 28-day | Age continuous, standardized | 1150 | 333 | 1.111 (0.999–1.235) | 0.052 |
| Zigong | 90-day | Original eight-category age as ordinal score | 1810 | 36 | 1.679 (1.119–2.520) | 0.012 |
| MIMIC-IV | 90-day | Age continuous, standardized | 1150 | 433 | 1.078 (0.984–1.182) | 0.107 |

HRs express the association per doubling of NPNR. Zigong publicly available age was reported in eight intervals; the ordered category score uses one model degree of freedom and is not an observed continuous age. MIMIC-IV provided numeric age, standardized for this sensitivity analysis. Other covariates matched each cohort’s original Model 3.

**Supplementary Table S9. Absolute mortality events by NPNR tertile in the Zigong cohort**

| **Dataset** | **NPNR tertile** | **N** | **Deaths** | **Percent (%)** |
| --- | --- | --- | --- | --- |
| 28-day fixed status | T1 | 629 | 3 | 0.48 |
| 28-day fixed status | T2 | 628 | 7 | 1.11 |
| 28-day fixed status | T3 | 628 | 22 | 3.50 |
| 90-day fixed status | T1 | 629 | 3 | 0.48 |
| 90-day fixed status | T2 | 628 | 10 | 1.59 |
| 90-day fixed status | T3 | 628 | 24 | 3.82 |
| 90-day Cox cohort | T1 | 629 | 3 | 0.48 |
| 90-day Cox cohort | T2 | 628 | 10 | 1.59 |
| 90-day Cox cohort | T3 | 627 | 23 | 3.67 |

NPNR tertiles are cohort-specific (T1–T3). One 90-day death in T3 lacked an exact event time and was excluded from 90-day Cox/Kaplan–Meier/spline analyses; this explains the difference between fixed-status and survival-analysis events. Complete-case event counts were unchanged.

**Supplementary Figure S1. Exploratory subgroup analyses of the association between NPNR and short-term all-cause mortality in the Zigong discovery cohort.**

(A) 28-day mortality. (B) 90-day mortality. Hazard ratios are expressed per doubling of NPNR. Interaction P-values were adjusted using the Benjamini-Hochberg false discovery rate procedure.

**Supplementary Figure S2. Cross-validation curves for Ridge Cox sensitivity analyses in the Zigong discovery cohort.**

(A) 28-day mortality. (B) 90-day mortality. NPNR was retained as the exposure of interest while the remaining covariates were ridge-penalized.

**Supplementary Figure S3. Comparison of Cox and Firth-penalized estimates in the Zigong discovery cohort.**

28-day and 90-day hazard ratios for each doubling of NPNR. Original and newly added models use identical complete-case datasets; Firth penalization was applied to Model 1 and the simplified core model, not to expanded Model 3.
